# Seroprevalence of Jamestown Canyon virus in the Japanese general population

**DOI:** 10.1186/s12879-020-05517-2

**Published:** 2020-10-23

**Authors:** Hirofumi Kato, Masaaki Satoh, Madoka Kawahara, Satoshi Kitaura, Tomoki Yoshikawa, Shuetsu Fukushi, Kristina Dimitrova, Heidi Wood, Masayuki Saijo, Mutsuyo Takayama-Ito

**Affiliations:** 1 Department of Virology 1, National Institute of Infectious Diseases, 1-23-1 Toyama, Shinjuku-ku, Tokyo, 162-8640 Japan; 2 Zoonotic Diseases and Special Pathogens Division, Public Health Agency of Canada, 1015 Arlington Street Winnipeg, Winnipeg, Manitoba R3E 3R2 Canada

**Keywords:** Jamestown canyon virus, Snowshoe hare virus, California serogroup, Seroepidemiology, Orthobunyavirus, Encephalitis, Japan

## Abstract

**Background:**

Jamestown Canyon virus (JCV) is a mosquito-borne orthobunyavirus that causes acute febrile illness, meningitis, and meningoencephalitis, mainly among adults. JCV is widely distributed in North America and the number of JCV cases in the U.S. has increased in recent years. Therefore, the central nervous system disease caused by JCV can be considered a potentially re-emerging viral disease. However, the seroprevalence of JCV is unknown in Japan. The purpose of this study is to evaluate the seroprevalence of JCV in the Japanese population.

**Methods:**

We used an IgG enzyme-linked immunosorbent assay (IgG-ELISA) with JCV-infected cell-lysates and/or a neutralizing (NT) antibody assay. The cut-off value of IgG-ELISA was determined using IgG-ELISA to analyze serum specimens from 37 healthy Japanese donors. IgG-ELISA was validated by assessing its sensitivity and specificity, using 38 human serum samples previously tested for the presence or absence of antibodies against JCV and snowshoe hare virus (SSHV), in an in-house NT antibody assay conducted by the Public Health Agency of Canada. The seroepidemiological study was performed using IgG-ELISA and NT antibody assay to analyze 246 human serum samples from the serum bank of the National Institute of Infectious Diseases (NIID) in Japan.

**Results:**

The cut-off value of IgG-ELISA was determined at 0.20, based on the mean (− 0.075) and standard deviation (0.092) values using Japanese donors’ sera. The sensitivity and the specificity of IgG-ELISA determined using 25 JCV-positive and 4 JCV-negative serum samples were 96 and 100%, respectively. Analysis of the 246 Japanese serum samples revealed that no specimen showed a higher value than the cut-off value of IgG-ELISA, and no sample tested positive by the NT antibody assay.

**Conclusions:**

Our results showed that JCV is not circulating significantly in Japan. To the best of our knowledge, this is the first report to demonstrate the seroprevalence of JCV in the general population in Japan.

## Background

Jamestown Canyon virus (JCV) belongs to the genus *Orthobunyavirus* in the family *Peribunyaviridae* of the order Bunyavirales [1]. JCV causes acute febrile illness and sometimes central nervous infections such as meningitis and meningoencephalitis, mainly among adults [2, 3]. JCV is classified into the California serogroup (CSG), along with La Crosse virus (LACV), snowshoe hare virus (SSHV), Inkoo virus (INKV), and Tahyna virus (TAHV) [4]. CSG viruses contain tri-segmented, negative sense RNA genomes [5]. The serogroup is worldwide prevalent and includes human pathogens such as LACV and SSHV in North America; INKV and TAHV in Asia and Europe; Guaroa virus in North and South America; and Lumbo virus in Africa [6]. CSG viruses use mosquito vectors, primarily species from the *Aedes* and *Ochlerotatus* genera. The mammalian host species include small rodents for SSHV, LACV, and TAHV; hares for SSHV, TAHV, and INKV; and deer for JCV [5, 7]. Currently, there are no available vaccines or specific treatments against CSG viruses.

JCV was first isolated from *Culiseta inornata* mosquitoes at Jamestown Canyon, Colorado, in the United States in 1961 [8]. JCV is widely prevalent in North America. Until 2012, 0–3 JCV cases had been reported in the U.S. However, this number increased to 75 and 41 cases in US northern states in 2017 and 2018, respectively [9, 10]. In addition, the ratio of symptomatic to asymptomatic individuals is speculated to be in the range of 1:100 to 1:1500 [11]. These data suggest that JCV can be considered a potentially re-emerging virus. The main vectors and reservoirs of JCV include mosquito species from the *Aedes*, *Coquillettidia*, *Culex*, and *Culiseta* genera [12, 13], and white-tail deer [14], respectively.

CSG viruses, including JCV, have not been isolated from mosquitoes or mammals in Japan. To date, no CSG infection cases have been reported in Japan. However, a nationwide study on mosquito distribution revealed that *Aedes*, *Culex*, and *Culiseta* are widely distributed in Japan [15]. Since deer, horses, and goats inhabit Japan and can act as reservoirs in the JCV lifecycle, there is a potential risk for the establishment of the JCV lifecycle in Japan. Mosquito-borne viral infections in humans have increased gradually worldwide, since global warming affects the geographical distribution and activity of mosquitoes. In fact, the 2015 dengue fever outbreak in Tokyo occurred as an autochthonous transmission of dengue virus, after 70 years from the last dengue fever outbreak in Japan after World War II [16]. Thus, mosquito-borne diseases became a public health threat in Japan and in disease-free areas. Although JCV infection cases have not been investigated to date, surveillance for mosquito-borne orthobunyavirus diseases is recommended.

To evaluate the prevalence of JCV in Japan, we developed an IgG enzyme-linked immunosorbent assay (IgG-ELISA) using JCV-infected cell-lysates as antigens and human serum specimens to conduct a serological survey among the residents of Japan.

## Methods

### Cells and viruses

Vero cells and Huh-7 cells were purchased from ATCC (# CCL-819) and BIKEN, Osaka University, respectively. All cells were grown in Dulbecco’s modified Eagle medium (DMEM; Sigma-Aldrich, St. Louis, MO) supplemented with 5% heat-inactivated fetal bovine serum (FBS; Biowest, Nuaille, France), non-essential amino acids (Sigma-Aldrich), and antibiotics (100 U/mL penicillin and 100 μg/mL streptomycin; Thermo Fisher Scientific, Waltham, MA). The JCV 61 V-2235 strain was purchased from ATCC (VR-712).

### Serum samples

Serum samples obtained from 37 healthy Japanese donors, which were negative in the NT assay, were used as negative controls to establish the cut-off value of IgG-ELISA. Forty human serum samples, which were stored at the Zoonotic Diseases and Special Pathogens Division, Public Health Agency of Canada, were tested for the presence or absence of JCV and SSHV, during an in-house neutralizing (NT) antibody assay conducted by the Public Health Agency of Canada [17]. Two additional specimens with an NT antibody titer against JCV equal to that against SSHV were excluded. The 38 specimens were classified into five groups as follows: group 1: JCV- and SSHV-positive, with an NT antibody titer against JCV 4-fold higher than that against SSHV (10 samples); group 2: JCV-positive and SSHV-negative (15 samples); group 3: JCV- and SSHV-positive, and an NT antibody titer against JCV inferior (4-fold) to that against SSHV (5 samples); group 4: JCV-negative and SSHV-positive (4 samples); group 5: JCV- and SSHV-negative (4 samples). Individuals from groups 1 and 2, 3 and 4, and 5 were diagnosed with JCV-positive, SSHV-positive, and negative, respectively. Several thousands of volunteers’ serum samples from all over Japan are stored in the serum bank of the National Institute of Infectious Diseases (NIID) in Japan when annual national epidemiological surveillance of vaccine-preventable diseases is performed. The serum samples stored in the serum bank were collected mostly from children, and has limited samples from adults, especially elderly people (more than 65 years old). Among these, serum samples used in this study were selected from those obtained and stored between 2011 and 2016. The proportions of age, sex, and regions of the blood donors were matched with the same proportions of the general population in Japan, based on the statistics published by the Statistics Bureau of Japan [18]. Sample size was calculated using Stata 13 software for Windows (StataCorp LP, College Station, TX), based on an estimated seroprevalence of 20% among the Japanese population with an interval width of 0.10 (upper 0.05, lower 0.05) and a confidential level of 95% [17], along with considering limited available samples the serum bank can prepared. According to the calculation, a total of 246 Japanese specimens from the serum bank were tested. All samples were inactivated at 56 °C for 30 min in a water bath.

### IgG-ELISA for detecting antibodies against JCV

JCV-positive and JCV-negative antigens were prepared as follows: Huh-7 cells infected with JCV at a multiplicity of infection of 0.05 and mock-infected Huh-7 cells were cultured for 2 days at 37 °C and 5% CO2 saturation in maintenance medium, consisting of DMEM containing 2% FBS (DMEM-2FBS). The virus- and mock-infected cells were washed with phosphate-buffered saline solution (PBS) and soaked for 20 min in PBS containing 1% Nonidet P-40. Lysates were centrifuged at 4000 rpm for 10 min. The supernatant fractions of the virus- and mock-infected cell solutions were used as JCV-positive and JCV-negative antigens, respectively. ELISA was performed as described elsewhere with minor modifications [19, 20]. Briefly, 96-well plates (Nunc-Immuno™ Plate, Thermo Fisher Scientific, Waltham, MA) were coated with JCV-positive and JCV-negative antigens at 4 °C overnight, then incubated at 18–25 °C for 2 h with heat-inactivated sera from the enrolled individuals, which were diluted 4-fold from 1:100 to 1:6400 as previously reported [21, 22]. Plates were washed thrice with 0.05% Tween-20 with PBS (PBST), then incubated with a 1:2000 diluted by blocking buffer of horseradish peroxidase (HRP)-conjugated goat anti-human IgG (goat anti-human IgG (H + L) cross-absorbed secondary antibody, HRP; Thermo Fisher Scientific) at 37 °C for 1 h. Plates were washed thrice with PBST and incubated with 100 μL of substrate solution (ELISA POD Substrate TMB Kit, Popular; Nacalai Tesque, Kyoto, Japan) at 18–25 °C for 4 min. The enzyme reaction was terminated using 100 μL of 1 M sulfuric acid. The absorbance (optical density, OD) was measured at a wavelength of 450 nm (OD_450_) using a microplate reader (iMark, Bio-Rad Laboratories Inc., Hercules, CA). The OD_450_ values of negative antigen wells were subtracted from the OD_450_ values from the corresponding positive antigen samples. The cut-off value was set as the average value plus three times standard deviation (SD) of the control sera from Japanese donors’ sera. Samples were considered IgG-ELISA positive if it yielded an OD_450_ value above the cut-off value.

### Neutralization assay

An NT assay was carried out as previously described with minor modifications [17]. Briefly, serum samples were heat-inactivated at 56 °C for 30 min, then diluted 10-fold with DMEM-2FBS. Subsequently, 75 μL of each dilution was mixed with a same volume of DMEM-2FBS containing 100 50% tissue culture infectious dose (TCID_50_) of JCV, then incubated at 37 °C for 1 h. Thereafter, 100 μL of these mixtures were used to inoculate Vero cell monolayers, then cultured at 37 °C for 5 days. After incubation, cells were fixed with 10% formalin for 1 h, then stained with methylene blue solution. After washing with distilled water, cytopathic effects were observed.

### Statistical analysis

The Mann–Whitney U test was used to compare OD_450_ values from IgG-ELISA in each group of human sera. The non-parametric Spearman rank correlation coefficient was used to evaluate the relationship between OD_450_ values and titers of JCV and SSHV NT antibodies. All *p*-values were two-sided, and *p* < 0.05 was considered significant; p-values were corrected by Bonferroni adjustments when multiple comparisons were implemented. All data were analyzed using GraphPad Prism 8 for Windows (GraphPad Software Inc., San Diego, CA).

## Results

### Determination of the cut-off value of IgG-ELISA using sera from Japanese healthy donors

The OD_450_ value of 4-fold serially diluted serum specimens from 37 Japanese donors was measured to determine the cut-off of IgG-ELISA. We found that the cut-off was 0.20, based on the mean (− 0.075) and SD (0.092) values.

### Determination of the sensitivity and specificity of the JCV-antigen-based IgG-ELISA

**Fig. 1:**
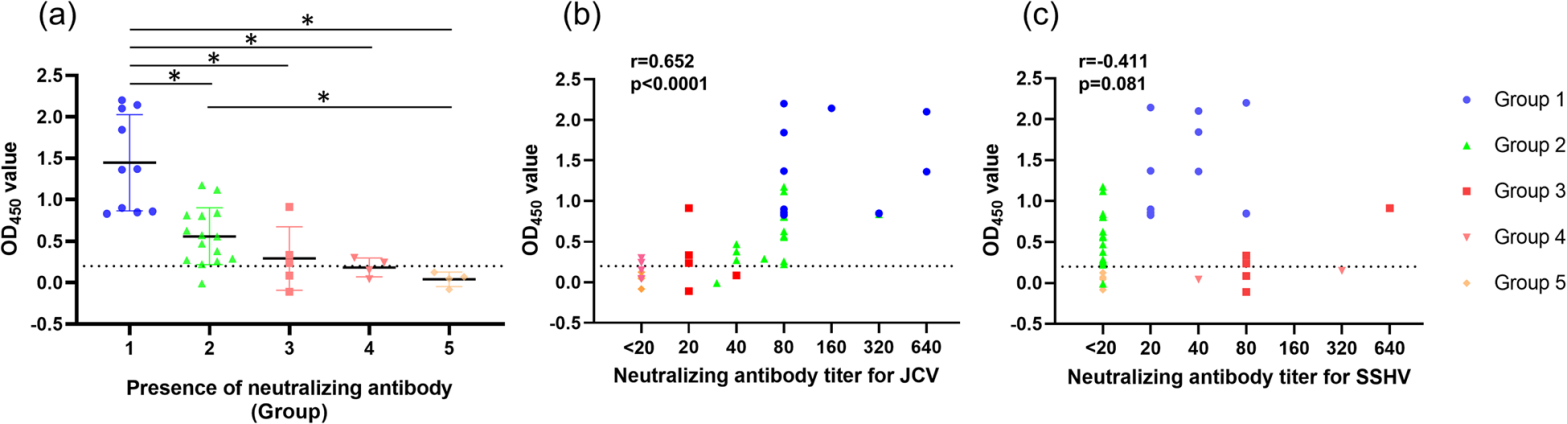
Comparison of IgG-ELISA and NT antibody assay for detecting JCV in human sera. **a** Spectrophotometric results for assessing the specificity and sensitivity of IgG-ELISA for detecting JCV in human sera. The 38 specimens were classified into 5 groups based on previous results; group 1: JCV and SSHV both-positive and NT antibody titer for JCV was higher than that for SSHV (10 samples); group 2: JCV-positive and SSHV-negative (15 samples); group 3: both-positive and NT antibody titer for JCV was less than that for SSHV (5 samples); group 4: JCV-negative and SSHV-positive (4 samples); group 5: both-negative (4 samples). **b**, **c** Comparison between IgG-ELISA OD_450_ values and neutralization antibody titers for JCV and SSHV. Statistical analyses were performed using the Mann–Whitney U test and Spearman’s rank correlation. Asterisks indicate *p* < 0.005

The sensitivity and specificity of IgG-ELISA were assessed using the 38 sera provided by the Public Health Agency of Canada (Fig. 1a). We found that 24 of the 25 specimens classified as group 1 or 2 (JCV-positive) tested positive by IgG-ELISA, whereas all 4 samples from group 5 (SSHV-negative) tested negative for JCV. Therefore, the sensitivity and specificity were calculated as 96 and 100%, respectively. On the other hand, 4 samples of the 9 specimens from groups 3 and 4 (SSHV-positive) tested positive for JCV, showing a 44% cross-reactivity with antibodies against SSHV. The OD_450_ values in groups 1 and 2 were significantly higher than those observed in the other groups and in group 5, respectively.

### Comparison between OD_450_ values from IgG-ELISA and neutralization antibody titers

To evaluate the relationship between IgG-ELISA and NT antibody titers, a statistical comparison was performed between OD_450_ values from IgG-ELISA and antibody titers from the neutralization test. Our results showed a nearly linear relationship between OD_450_ values and NT antibody titers against JCV (Spearman’s correlation coefficient of 0.652 (*p* < 0.0001)) (Fig. 1b). In contrast, no correlation was found between OD_450_ values and NT antibody titers against SSHV (Spearman’s correlation coefficient of − 0.411 (*p* = 0.081)) (Fig. 1c).

### Seroprevalence of antibodies against JCV among the Japanese population

The number and characteristics of samples (age, sex, and regions) are shown in Table 1. A total of 246 Japanese specimens from the serum bank were tested using IgG-ELISA and NT antibody assay. No sample showed a positive reaction by IgG-ELISA or NT assay.

**Table 1:** Number and characteristics of samples by age and regions

Region	Hokkaido/Tohoku	Kanto	Chubu	Kinki	Chugoku	Shikoku	Kyushu	Total
Age (years)								
0–19	6	14	8	8	4	2	4	46
20–39	6	18	8	10	4	2	8	56
40–59	8	22	10	12	4	2	8	66
60-	8	26	14	14	4	2	10	78
Total	28	80	40	44	16	8	30	246

Specimens were provided by the serum bank of the National Institute of Infectious Diseases in Japan. The number of males and females is equal. The main cities (latitude/longitude) in each region are Sapporo/Sendai (43° 03′ 51″ N, 141° 20′ 49″ E / 38° 16′ 08″ N, 140° 52′ 19″ E) in Hokkaido/Tohoku, Tokyo (35° 41′ 22″ N, 139° 41′ 30″ E) in Kanto, Nagoya (35° 10′ 49″ N, 136° 54′ 24″ E) in Chubu, Osaka (34° 41′ 11″ N, 135° 31′ 12″ E) in Kinki, Hiroshima (34°23′47″ N, 132° 27′ 34″ E) in Chugoku, Takamatsu (34° 20′ 25″ N, 134° 02′ 36″ E) in Shikoku, and Fukuoka (33° 36′ 23″ N, 130° 25′ 05″ E) in Kyushu

## Discussion

Humans get infected with JCV primarily through the mosquito bite belonging to the genera *Ochlerotatus, Aedes, Coquillettidia, Culex,* and *Culiseta* [5, 7]. Mammalian host species such as white-tailed deer, mule deer, sika deer, sheep, horses, goats, etc. serve as reservoirs for the virus [6]. Since deer, horses, and goats that inhabit Japan can act as reservoirs in the JCV lifecycle, there is a potential risk of JCV spreading in Japan. A study conducted in Nova Scotia, Canada, using human sera for unrelated diagnosis, revealed a seropositivity of 21.2% for JCV [17]. Additionally, in a seroprevalence study conducted in Quebec, Canada, 9–24% tested individuals were positive for JCV [23]. On the basis of these surveys, it is estimated that one fourth of the population in endemic areas has antibodies against JCV. Conversely, the results from our study using JCV-antigen-based IgG-ELISA with a high sensitivity and specificity showed that none of the 246 analyzed samples from Japan tested positive, suggesting that JCV infection does not exist in Japan at least as frequently as in some endemic areas, given the number of samples tested.

In China, TAHV was isolated from *Culex* spp. mosquitoes collected in the Xinjiang Uygur Autonomous Region, and TAHV antibodies were detected in human samples [24]. Accordingly, we first speculated that CSG viruses might have spread widely in East Asian countries, including Japan. CSG viruses are known to exhibit serological cross-reactivity [25]. Additionally, the detection of anti-JCV IgM using ELISA may indicate infection by other CSG viruses [2, 26]. The JCV-IgG ELISA developed in this study showed only a partial cross-reactivity (44%) with antibodies against SSHV. Although cross-reactivity against only SSHV was investigated in this study, further evaluations on cross-reactivity against other CSG viruses are also required in the JCV-IgG ELISA. Considering the seroepidemiological results, imported cases of CSG viruses into Japan should be monitored. It is noteworthy that infection with CSG viruses should be considered as a differential diagnosis in patients returning from endemic areas, with a history of mosquito-bite and unknown encephalitis. Hereafter, an early outbreak detection system based on a risk assessment approach would be required, by implementing a surveillance program for detecting antibodies against JCV in animal sera and mosquitoes.

In this study, JCV-infected cell-lysates were used as antigens for IgG-ELISA. It has been reported that the sensitivity and specificity of ELISA for flaviviruses from Vero cell-lysates were lower than those observed using a recombinant protein-based ELISA, possibly due to impurities from cell-lysates [27]. However, the JCV-antigen IgG-ELISA showed high sensitivity and specificity. Additionally, we evaluated the presence of antibodies against JCV using an NT antibody assay. No Japanese serum sample tested positive by ELISA or NT antibody assay. Based on these data, we consider that the possibility of a wide distribution of JCV in Japan is lower than initially hypothesized.

Notably, the OD_450_ values of samples from groups 1 and 2 tested by IgG-ELISA were higher than those found in samples from groups 3 and 4 diagnosed with SSHV. Besides, we found a nearly linear relationship between OD_450_ values and NT antibody titers against JCV. Since the OD_450_ values can increase owing to the presence of NT antibodies against SSHV, IgG-ELISA might not be as efficient as the NT antibody test in distinguishing between JCV and SSHV completely, especially at border line from 0.2 to 1.0 of the OD_450_ values. Nevertheless, IgG-ELISA can be used to diagnose JCV without a neutralizing assay when OD_450_ values shows more than 1.0.

A limitation of this study could be a biased selection of samples because the samples from the serum bank of NIID were provided by volunteers and not selected by random sampling. Furthermore, it is possible that the true prevalence of JCV might be less than the detection limit because of the small number of samples used in the current study. Although several thousands of volunteers’ serum samples have been stored in the serum bank of NIID, we could not freely use these samples because of finite scientific resources. Moreover, the serum bank has limited samples from adults, especially elderly people (more than 65 years old), since most samples have been collected from children to investigate the prevalence of vaccine-preventable diseases.

## Conclusions

Our results showed that JCV is not circulating significantly in Japan. To the best of our knowledge, this is the first report demonstrating JCV seroprevalence in the general population in Japan.

## Data Availability

The datasets used and analysed during the current study are available from the corresponding author on reasonable request.

## Abbreviations

ELISA: Enzyme-linked immunosorbent assay
IgG: Immunoglobulin G
FBS: Fetal bovine serum
DMEM: Dulbecco’s modified eagle medium
ATCC: American type culture collection
NT: Neutralizing
NIID: National institute of infectious diseases
OD: Optical density
SD: Standard deviation
CSG: California serogroup
JC: Jamestown Canyon
LACV: La Crosse virus
SSHV: Snowshoe hare virus
INKV: Inkoo virus
TAHV: Tahyna virus
